# Association of Modifiable Lifestyle Factors With Cortical Amyloid Burden and Cerebral Glucose Metabolism in Older Adults With Mild Cognitive Impairment

**DOI:** 10.1001/jamanetworkopen.2020.5719

**Published:** 2020-06-09

**Authors:** Noriyuki Kimura, Yasuhiro Aso, Kenichi Yabuuchi, Masato Ishibashi, Daiji Hori, Yuuki Sasaki, Atsuhito Nakamichi, Souhei Uesugi, Mika Jikumaru, Kaori Sumi, Atsuko Eguchi, Hitoshi Obara, Tatsuyuki Kakuma, Etsuro Matsubara

**Affiliations:** 1 Department of Neurology, Faculty of Medicine, Oita University, Oita, Japan; 2 Biostatics Center, Kurume University, Kurume, Japan

## Abstract

**Importance:**

Understanding the association of lifestyle factors with mild cognitive impairment enables the development of evidence-based interventions for delaying cognitive impairment.

**Objective:**

To explore whether objectively measured lifestyle factors, such as physical activity, conversation, and sleep, are associated with cortical amyloid burden and cerebral glucose metabolism in older adults with mild cognitive impairment.

**Design, Setting, and Participants:**

This cohort study included 855 community-dwelling adults in Usuki, Oita Prefecture, Japan, aged 65 years or older. Data were collected from August 2015 to December 2017. Participants were reviewed to examine risk and protective lifestyle factors for dementia. Data analysis was conducted in June 2019.

**Exposures:**

Wearable sensors, carbon-11 labeled Pittsburgh compound B positron emission tomography images, and fluorine-18 fluorodeoxyglucose positron emission tomography images.

**Main Outcomes and Measures:**

Wearable sensor data, such as walking steps, conversation time, and sleep, were collected from August 2015 to October 2017, and positron emission tomography images were collected from October 2015 to December 2017. A multiple regression model and change-point regression model were used to examine the association of lifestyle factors with mean amyloid or fluorodeoxyglucose uptake, assessed on the basis of a standardized uptake value ratio of the frontal lobes, temporoparietal lobes, and posterior cingulate gyrus with the cerebellar cortex as the reference region. The bootstrap method was used to obtain nonparametric 95% CIs on the associations of lifestyle factors with cognitive decline.

**Results:**

Of the 855 adults in the study, 118 (13.8%) were diagnosed with mild cognitive impairment, with a mean (SD) age of 75.7 (5.8) years and 66 (55.9%) women. Total sleep time was inversely associated with fluorodeoxyglucose uptake after adjusting for covariates (β = −0.287; 95% CI, −0.452 to −0.121, *P* < .001). Change-point regression showed an inverse association between total sleep time and mean amyloid uptake when sleep duration was longer than 325 minutes (B = −0.0018; 95% CI, −0.0031 to −0.0007).

**Conclusions and Relevance:**

To our knowledge, this is the first study to demonstrate that total sleep time was associated with brain function in older adults with mild cognitive impairment. Sleep duration is a potentially modifiable risk factor for dementia at the mild cognitive impairment stage.

## Key Points

**Question:**

Are modifiable lifestyle factors associated with cortical amyloid burden or cerebral glucose metabolism in older adults with mild cognitive impairment?

**Findings:**

This cohort study included 118 older adults with mild cognitive impairment and found that total sleep time was associated with cerebral glucose metabolism after adjusting for covariates and false-discovery rate correction.

**Meaning:**

In this study, the association of sleep duration with brain function was confirmed in older adults with mild cognitive impairment, thereby strengthening the potential of sleep as an evidence-based intervention for preventing cognitive impairment.

## Teaser

This cohort study explores whether objectively measured lifestyle factors, such as physical activity, conversation, and sleep, are associated with cortical amyloid burden and cerebral glucose metabolism in older adults with mild cognitive impairment.

## Introduction

Mild cognitive impairment (MCI) is a high-risk factor for dementia^1^ and a major public health issue worldwide, posing a serious social and financial burden on patients and caregivers.^2^ Although the prevalence of late-life dementia is expected to increase, effective disease-modifying treatments are currently unavailable. Therefore, it is important to elucidate risk or protective factors associated with the cortical amyloid burden or brain function to delay cognitive impairment. Potentially modifiable risk factors for dementia have been identified and include low levels of education, vascular risk factors, lifestyle factors, and hearing loss.^2,3,4,5^ In particular, physical inactivity, sleep disturbance, social isolation, and depression are important risk factors for late-life cognitive impairment.^5,6,7,8^ Most observational studies have used self-report questionnaires that are of limited utility owing to their poor reliability and consistency, which is attributed to recall bias or misclassification, particularly among older people or those with MCI.^8,9^ Wearable sensors have been used to evaluate lifestyle factors, such as physical activity and sleep, in large epidemiological studies.^10,11,12,13,14^ Wearable sensors are noninvasive and cost-effective and can objectively measure total daily movement and sleep without recall bias. We have previously reported an association between objectively measured walking steps, conversation time, and global cognitive function in community-dwelling older adults,^14^ based on which we hypothesized that these lifestyle factors were associated with cortical amyloid burden or brain function. To our knowledge, only a few studies have examined the association between objectively measured lifestyle factors and the results of positron emission tomography (PET) imaging, including carbon-11 labeled Pittsburgh compound B (PiB)-PET and fluorine-18 fluorodeoxyglucose (FDG)-PET in older adults with MCI. Therefore, the present study aimed to use wearable sensors to explore whether modifiable lifestyle factors are associated with cortical amyloid burden and cerebral glucose metabolism in community-dwelling older adults with MCI.

## Methods

### Ethics

This prospective cohort study was conducted in accordance with the Declaration of Helsinki^15^ and approved by the local ethics committee of Oita University Hospital. Written informed consent was obtained from all participants. This study followed the Strengthening the Reporting of Observational Studies in Epidemiology (STROBE) reporting guideline.

### Participants

This was a prospective cohort study initiated in 2015 that examined risk and protective lifestyle factors for dementia among older adults in Usuki, Oita Prefecture, Japan. Adults aged 65 years and older account for approximately 38% of the Usuki population. Public servants recruited adults without dementia via public relations initiatives. The inclusion criteria were as follows: (1) aged 65 years and older; (2) living in Usuki; (3) physically and psychologically healthy; (4) without dementia; and (5) independent function in activities of daily living. Participants were required to wear a wristband sensor for a mean of 7 to 14 days per measurement period. Measurement of lifestyle factors repeated every 3 months for 1 year (ie, 4 times per year for 56 days during the study period) to eliminate measurement errors owing to seasonal differences in lifestyle.^16^ All participants underwent physical examination and cognitive function evaluation using the Mini-Mental State Examination and medical interview at baseline. Height and weight were measured, and body mass index was calculated as weight in kilograms divided by height in meters squared. We collected information on patient demographic characteristics, including age, sex, education level, smoking status, alcohol consumption, and medication history, via interviews conducted by trained medical staff at baseline. Assessment of vascular risk factors, such as hypertension, diabetes, and hypercholesterolemia, were based on clinical history and medication (antihypertensive, antidiabetic, or anticholesterol medication) use. Between August 2015 and October 2017, 855 community-dwelling adults (538 [62.9%] women; mean [SD] age, 73.8 [5.8] years, median [interquartile range {IQR}] education level 12 [11-12] years) satisfied the criteria and had valid sensing data for analysis.^14^ Of 855 adults, 118 (13.8%) who were diagnosed with MCI and received PiB-PET and FDG-PET were recruited to this study. The diagnosis of MCI was made according to previous studies,^17^ as follows: (1) subjective memory complaints and objective memory impairment, (2) Clinical Dementia Rating score of 0.5, and (3) absence of significant impairment in cognitive function or activities of daily living.

### Wearable Sensor Data

All participants wore a wristband sensor (Silmee W20, TDK Corporation) day and night except while bathing. The wearable sensor measured various lifestyle parameters, including walking steps, conversation time, and various sleep parameters, as described previously.^14^ These parameters were calculated by summing the sensor data collected each day and averaging it for the measurement period. Walking steps were calculated by averaging the total number of steps per day. Sleep parameters, such as total sleep time (TST), sleep efficiency, time awake after sleep onset (WASO), and waking count were measured from 6:00 pm to 5:59 am the following morning; start was defined as the clock time associated with the beginning of the first 20-minute block of sleep without movement. Total sleep time was the average total number of minutes slept per day. Sleep efficiency was calculated as the percentage of TST during the time spent in bed. Nocturnal waking was defined as 20 minutes of continuous movement from sleep onset to the end of sleep. Time awake after sleep and waking time count were calculated by averaging the total number of minutes awake after sleep onset and frequency of waking per day, respectively. Nap time was defined as a resting period without movement on the wearable sensor from 6:00 am to 5:59 pm. The sensor could determine whether adults and those nearby made utterances. Although the utterances of other individuals were included in the sound data, participation in the conversation was considered an important as a measure of social activity. Sound data, but not the content of conversations, were continuously collected every minute via a microphone on the wearable sensor and analyzed to evaluate the amount of time spent engaged in conversation. The wearable sensor detected sound pressure levels for utterances that originated within a 2-meter radius from the device. The sound pressure level ranged from 55 to 75 A-weighted decibels at this distance. A frequency band corresponding to a human voice from the sound data within the sound pressure range was extracted as a sound frame. A conversation was defined as a period of 1 minute with more than 4 sound frames. We verified the measurement accuracy for walking steps, conversation time, and sleep time by comparing the sensor data with video observation data in healthy older adults.^14^

### Imaging Data Acquisition

Static carbon-11 labeled PiB-PET and fluorine-18 FDG-PET findings were acquired using the Biograph mCT (Siemens) in 3-dimensional scanning mode, as previously described.^18,19^ For PiB-PET, a bolus of carbon-11 labeled PiB (mean [SD], 555 [185] MBq) was intravenously injected, followed by a saline flush and scanning for 20 minutes, starting 50 minutes after injection. Radioactivity concentration was measured during 50 to 70 minutes after injection. For FDG-PET, all participants rested with their eyes closed for 10 minutes in a dimly lit room before the injection. Subsequently, a bolus of flourine-18 FDG (3.0 MBq/kg) was intravenously injected, followed by a saline flush and scanning for 20 minutes, starting 40 minutes after injection. For FDG-PET, radioactivity concentration was measured during 40 to 60 minutes after injection. The injected dose for each participant was confirmed by measuring the radiation in predose and postdose samples using a radiation detector. All imaging data were reconstructed into a 3.0-mm thick slice, a 256 × 256 matrix, and magnification ×3.0, with an ordered subset expectation maximization, which included 4 iterations and 12 subsets. Reconstructed images had a pixel size of 1.06 mm. Both PiB and FDG scans were spatially normalized to a customized PET template at the Montreal Neurological Institute reference space, using Statistical Parametric Mapping version 8 (Wellcome Trust Centre for Neuroimaging). The MarsBaR (MRC Cognition and Brain Sciences Unit) region of interest (ROI) toolbox for Statistical Parametric Mapping was used to define ROIs for the frontal lobes, temporoparietal lobes, posterior cingulate gyrus, and cerebellum, as described previously.^20^ These ROIs included those with amyloid deposition detected using amyloid PET or those with decreased cerebral glucose metabolism detected using FDG-PET in patients with Alzheimer disease.^21,22^ The ROI values were averaged across both hemispheres. We assessed PiB and FDG uptake on the basis of a standardized uptake value ratio (SUVR), which was calculated from the voxel number–weighted average of the median uptake in the frontal, temporoparietal, and posterior cingulate ROIs in reference to the ROI in the cerebellum. The mean cortical SUVR in FDG-PET or PiB-PET was represented as the single mean value for all the regions combined.

### Apolipoprotein E Phenotype

Apolipoprotein E (*APOE*) phenotype determination was performed using Human Apolipoprotein E4/Pan-*APOE* ELISA Kit (MBL Co), which measures the amount of *APOE *ε4 (*APOE4*) or total *APOE* specifically with high sensitivity using affinity-purified polyclonal antibody against *APOE* and monoclonal antibody against *APOE4* by sandwich ELISA. It can also measure the differences among the homozygotes (ie, ε4/ε4) and heterozygotes (ε2/ε4, ε3/ε4) of *APOE4* phenotypes and non-*APOE4* zygotes (ε2/ε2, ε3/ε3, and ε2/ε3) by the ratio of *APOE* and *APOE4* levels.^23,24,25^

### Statistical Analysis

The association between neuroimaging variables and 7 lifestyle factor variables (walking steps, conversation time, TST, WASO, sleep efficiency, waking time count, and nap time) was examined with the following methods. First, a multiple regression model was performed to examine the association between the 7 lifestyle factor variables and mean PiB-PET or FDG-PET uptake, after adjusting for covariates, including age, sex, education level, *APOE4* status, body mass index, vascular risk factors, alcohol consumption, and smoking status. A 2-tailed *P* < .05 was considered statistically significant. The resulting *P *values were corrected according to the false-discovery rate. Second, a smoothing spline curve was fitted to examine patterns of association between neuroimaging variables and lifestyle variables graphically. By visual inspection of a smooth spline curve, a simple spline regression model with 1 knot, referred to as a change-point model, was used to approximate nonlinear relationships. Then, the covariates were added to the change-point model. Overall, 2500 bootstrap sample data sets were generated to construct nonparametric confidence intervals of lifestyle factor parameters. Statistical analyses were conducted using SPSS statistical software version 25.0 (IBM Corp) and JMP Pro version 14.2.0 (SAS Institute).

## Results

### Clinical and Demographic Characteristics

Table 1 summarizes the sociodemographic factors, cognitive function, and lifestyle factors of the participants. The mean (SD) age was 75.7 (5.8) years, 52 (44.1%) were men, and 66 (55.9%) were women. The median (IQR) education level was 12 (9-12) years, and the median (IQR) Mini-Mental State Examination score was 26 (25-28) points. The mean (SD) duration of collecting lifestyle data from the participants was 31.3 (7.1) days per year (a mean of 7.8 days every 3 months) using the wristband sensor. The median (IQR) duration between PET study and initial wearing a wristband sensor was 71 (47-167) days for PiB-PET and 65 (42-166) days for FDG-PET. Walking steps and TST were similar to those reported in previous studies involving older Japanese adults.^26,27^ Based on a PiB-PET SUVR cutoff of 1.4, 27 adults (22.9%) were included in the amyloid-positive subgroup. Moreover, 15 adults (12.7%) showed abnormal PiB retention levels in more than 1 ROI, regardless of amyloid subgroup. The quantitative analysis of PiB-PET based on a SUVR cutoff of 1.4 cannot identify MCI adults with low amyloid accumulation, indicating early stage of MCI.

**Table 1.** Clinical and Demographic Characteristics

Characteristic	No. (%)
Age, mean (SD), y	75.7 (5.8)
Sex	
Men	52 (44.1)
Women	66 (55.9)
Education level, median (IQR), y	12 (9-12)
BMI, mean (SD)	23.2 (3.3)
MMSE score, median (IQR)	26 (25-28)
*APOE4*	17 (14.4)
Ever smoked	5 (4.2)
Ever drank	42 (35.6)
Medical history	
Hypertension	67 (56.8)
Diabetes	24 (20.3)
Hypercholesterolemia	37 (31.4)
Wearable sensor data	
Walking steps, mean (SD), steps/d	4610.5 (2558.4)
Conversation time, median (IQR), min/d	206.9 (148.0-259.7)
TST, mean (SD), min/d	403.7 (69.2)
WASO, median (IQR), min/d	23.3 (13.3-33.0)
Sleep efficiency, mean (SD), %/d	94.1 (3.4)
Waking time count, median (IQR), counts/d	0.52 (0.34-0.83)
Nap time, median (IQR), min/d	38.4 (21.2-64.1)
Global uptake from neuroimaging data, SUVR	
PiB, median (IQR)	0.93 (0.83-1.33)
FDG, mean (SD)	0.92 (0.08)

Abbreviations: *APOE4*, apolipoprotein E ε4; BMI, body mass index (calculated as weight in kilograms divided by height in meters squared); FDG, fluorine-18 F-fluorodeoxyglucose; IQR, interquartile range; MMSE, Mini-Mental State Examination; PiB, carbon-11 labeled Pittsburgh compound B; SUVR, standardized uptake value ratio; TST, total sleep time; WASO, time awake after sleep.

### Multiple Regression Model and Change-Point Regression Model

Table 2 summarizes the results of the multiple regression model after adjusting for covariates between lifestyle factors and mean PiB uptake. There were no significant associations between the 7 lifestyle factors and mean PiB uptake in the multiple regression model. However, after adjusting for covariates, the change-point regression model showed an inverse association between TST and mean PiB uptake when TST was longer than 325 minutes (B = −0.0018; 95% CI, −0.0031 to −0.0007) (Figure, A and Table 3). The inclination of the graph began to reverse by the boundary of the specified threshold (ie, 325 minutes) for TST, indicating the association between TST and PiB uptake when sleep duration was longer than 325 minutes. Results of the multiple regression model after adjusting for covariates between lifestyle factors and mean FDG uptake are shown in Table 4. A significant association was found between TST and mean FDG uptake after adjusting for covariates and correcting for the false-discovery rate (β = −0.287; 95% CI, −0.452 to −0.121, *P* < .001) (Figure, B). After adjusting for covariates, the change-point regression model showed no significant association with walking steps, conversation time, or sleep efficiency.

**Table 2.** Multiple Regression Model of Association of Lifestyle Factors With Cortical Amyloid Burden

Lifestyle factor	PiB mean SUVR	
	β (95% CI)	*P* value
Walking steps, steps/d	−0.044 (−0.228 to 0.14)	.64
Conversation time, min/d	−0.004 (−0.184 to 0.176)	.97
TST, min/d	−0.152 (−0.325 to 0.021)	.08
WASO, min/d	0.101 (−0.081 to 0.282)	.27
Sleep efficiency, %/d	−0.143 (−0.322 to 0.036)	.12
Waking time count, counts/d	0.11 (−0.076 to 0.295)	.24
Nap time, min/d	−0.033 (−0.204 to 0.138)	.70

Abbreviations: PiB, carbon-11 Pittsburgh compound B; SUVR, standardized uptake value ratio; TST, total sleep time; WASO, time awake after sleep.

**Figure.**
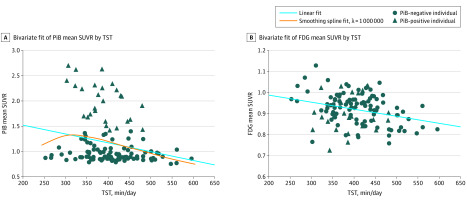
Association of Total Sleep Time (TST) With Positron Emission Tomography Imaging A, TST was inversely associated with mean carbon-11 labeled Pittsburgh compound B (PiB) uptake in the change-point regression model after adjusting for covariates. The inclination of the graph started to reverse because of the boundary of the specified threshold (ie, 325 minutes) for TST. TST was inversely associated with PiB uptake in sleep durations longer than 325 min. B, TST was inversely associated with mean fluorine-18 fluorodeoxyglucose (FDG) uptake in the multiple regression model after adjusting for covariates and the false-discovery rate correction. SUVR indicates standardized uptake value ratio.

**Table 3.** Change-Point Regression Model of Association of Lifestyle Factors With Cerebral Glucose Metabolism and Cortical Amyloid Burden

Lifestyle factor	B (knot)	Bootstrap 95% CI
**PiB mean SUVR**		
TST, min/d	−0.0018 (325)	−0.0031 to −0.0007
**FDG mean SUVR**		
Walking steps, steps/d	0.00001 (10000)	−0.0002 to 0.0001
Conversation time, min/d	0.0001 (150)	−0.0001 to 0.0003
Sleep efficiency, %/d	−0.0037 (92)	−0.0096 to 0.003

Abbreviations: FDG, fluorine-18 fluorodeoxyglucose; PiB, carbon-11 labeled Pittsburgh compound B; SUVR, standardized uptake value ratio; TST, total sleep time.

**Table 4.** Multiple Regression Model of Association of Lifestyle Factors With Cerebral Glucose Metabolism

Lifestyle factor	FDG mean SUVR	
	β (95% CI)	*P* value
Walking steps, steps/d	0.098 (−0.084 to 0.28)	.29
Conversation time, min/d	0.183 (0.007 to 0.358)	.04
TST, min/d	−0.287 (−0.452 to −0.121)	<.001
WASO, min/d	−0.176 (−0.354 to 2.6 × 10^−4^)	.05
Sleep efficiency, %/d	0.079 (−0.101 to 0.258)	.39
Waking time count, counts/d	−0.206 (−0.387 to −0.025)	.03
Nap time, min/d	−0.125 (−0.293 to 0.043)	.14

Abbreviations: FDG, fluorine-18 fluorodeoxyglucose; SUVR, standardized uptake value ratio; TST, total sleep time; WASO, time awake after sleep.

## Discussion

We examined the association between objectively measured lifestyle factors and PET imaging using multiple regression and change-point regression models. While several studies have individually examined the association between physical activity, sleep, or cognitive activity and AD biomarkers among cognitively healthy adults,^28,29,30,31,32,33,34,35,36^ to our knowledge, the present study is the first to clarify the association of various lifestyle factors with PET imaging simultaneously in older adults with MCI. In the present study, the number of adults with MCI and abnormal levels of PiB retention was relatively small compared with that reported in other studies,^37,38^ indicating a heterogeneous background pathology. A possible explanation for this discrepancy is the inclusion of MCI adults with non–Alzheimer disease pathology, such as Lewy body disease, transactive response DNA binding protein 43, argyrophilic grains, and hippocampal sclerosis.^39^ However, our results provide novel and interesting insights into the mechanisms underlying the association between lifestyle factors and cortical amyloid burden or brain function in older adults with MCI. First, TST was inversely associated with cortical amyloid burden among participants whose sleep duration was longer than 325 minutes. Second, the association of TST with cerebral glucose metabolism remained significant after adjusting for covariates and correcting for the false-discovery rate. The present study has several strengths, such as including adults with MCI selected from community-dwelling older individuals, objectively measuring various lifestyle factors, and assessing cortical amyloid burden or brain function by PET imaging.

The most interesting finding of the present study was that TST was inversely associated with cerebral glucose metabolism. Few studies have examined the association between sleep characteristics and FDG-PET in older adults. In the present study, the multiple regression model showed an inverse association between TST and cerebral glucose metabolism in older adults with MCI. Several cross-sectional or prospective studies have reported that a long sleep duration may be a risk factor for subsequent cognitive impairment.^7,40^ However, mechanisms underlying the association of longer sleep duration and dementia remain unclear. Longer sleep duration may be associated with sleep disorder–related breathing and smoking habits.^7^

Although the association of TST with mean PiB uptake was not significant in the multiple regression model, the change-point regression model showed that TST was associated with mean PiB uptake when sleep duration was longer than 325 minutes. This finding may support the potential mechanisms linking sleep and cortical amyloid burden in the previous studies.^34,35,36,40,41,42^ The potential mechanisms linking sleep and cortical amyloid burden have been identified in human and mouse models of Alzheimer disease.^34,35,36,40,41,42^ Short sleep time, poor sleep efficiency, long sleep latency, and frequent napping were associated with amyloid pathology in cognitively healthy older adults.^34,35^ Soluble β-amyloid was released with physiologic synaptic activity during wakefulness in a mouse model, with β-amyloid clearance being the highest during sleep.^41,42^ In younger individuals, diurnal fluctuations in brain interstitial fluid and cerebrospinal fluid^43^ indicate that chronic sleep deprivation may increase β-amyloid production and reduce β-amyloid clearance, leading to amyloid plaque formation.^43^ These results lead us to hypothesize that sleep duration may be an important lifestyle factor associated with cortical amyloid burden and brain function.

Few studies have examined the association of conversation time and PET imaging. We have previously reported the association between conversation time and cognitive function in older adults.^14^ However, conversation time was not associated with cortical amyloid burden or cerebral glucose metabolism in this study. Moreover, objectively measured walking steps were not associated with cortical amyloid burden or cerebral glucose metabolism. Previous studies,^44,45^ using an accelerometer, showed that moderately intense physical activity, but not light intensity or vigorous intensity physical activity, was favorably associated with cerebrospinal fluid β-amyloid 42 and cerebral glucose metabolism. These results support our findings because walking is considered a light intensity physical activity.

### Limitations

The present study has several limitations that should be considered. First, the study could not determine the causal direction of the association between lifestyle factors and cortical amyloid burden or brain function because of its cross-sectional design. Second, the background noise from television or radio might have been detected as conversation by the detection method, which was based on sound pressure level and frequency. Moreover, conversation time may have included sleep or nap time during television viewing or radio listening. However, according to a previous study,^14^ the possibility of including sleeping in the daily conversation time was only 6.4%. Third, we collated clinical data to define the presence or absence of dementia, but patients with possible dementia could not be completely excluded from the study. Fourth, the lack of association among walking steps, sleep time, and cortical amyloid burden might be because of a small number of adults with significant PiB uptake in our cohort. Therefore, this study must be considered preliminary, and further studies with larger sample size with cortical amyloid burden are required.

## Conclusions

To our knowledge, this is the first study to confirm the association of sleep duration with cortical amyloid burden and brain function in older adults with MCI. Sleep duration was inversely associated with cerebral glucose metabolism. Moreover, sleep duration was inversely associated with cortical amyloid burden among participants whose sleep duration was longer than 325 minutes. These results may contribute to the development of novel evidence-based interventions for delaying cognitive impairment in older adults.
